# Thromboelastometric assessment of hemostasis following hydroxyethyl starch (130/0.4) administration as a constant rate infusion in hypoalbuminemic dogs

**DOI:** 10.1186/s12917-018-1357-8

**Published:** 2018-01-31

**Authors:** Angelica Botto, Barbara Bruno, Cristiana Maurella, Fulvio Riondato, Alberto Tarducci, Giulio Mengozzi, Antonio Borrelli

**Affiliations:** 1 Department of Veterinary Science, University of Turin, Largo Paolo Braccini n°2-5, 10095 Grugliasco, TO Italy; 2 Istituto Zooprofilattico Sperimentale del Piemonte, Liguria e Valle d’Aosta, via Bologna 148, 10154 Torino, Italy; 3 Department of Public Health and Pediatric Sciences, C.so Bramante 88/90, 10100 Torino, Italy

**Keywords:** Dogs, Hydroxyethyl starch, Hypoalbuminemia, Thromboelastometry

## Abstract

**Background:**

The primary aim was to evaluate by means of thromboelastometry (ROTEM) the effects of hydroxyethyl starch (HES) 130/0.4 administered as a constant rate infusion (CRI) on hemostasis in hypoalbuminemic dogs. The second aim was to use ROTEM analysis to detect whether all hypoalbuminemic dogs of our population were hypercoagulable.

**Results:**

The study sample was 20 hypoalbuminemic dogs (albumin < 2 g/dl) with normal perfusion parameters and requiring intravenous fluid therapy. In order to support plasma colloid osmotic pressure, in addition to crystalloid, HES 130/0.4 was administered as a constant rate infusion at 1 ml/kg/h (group 1, *n* = 11) or 2 ml/kg/h for 24 h (group 2, *n* = 9). Blood samples were collected at baseline (T0) and 24 h postinfusion (T1); coagulation was assessed by standard coagulation profile (prothrombin time, activated partial thromboplastin time, and fibrinogen), and ROTEM analysis (in-TEM®, ex-TEM® and fib- TEM® profile).

No statistically significant differences in ROTEM values in group 1 were observed (*P* > 0.05), whereas in group 2 statistically significant differences (*P* < 0.05) were found at T1 in the in-TEM® profile [decrease in clot formation time (*P* = 0.04) and increase in α angle (*P* = 0.02)] and in the ex-TEM® profile [increase in maximum clot firmness (*P* = 0.008) and α angle (*P* = 0.01)]; no changes were identified in the fib-TEM® profile. In both groups, a statistically significant decrease (*P* = 0.007) in hematocrit was noted, whereas no statistically significant differences in platelet count and standard coagulation profile were found. In group 2, a statistically significant increase in TS values (*P* = 0.03) was noted at T1. ROTEM tracings indicating a hypercoagulable state were observed in 7/20 dogs at T0 (5/11 in group 1 and 2/9 in the group 2).

**Conclusion:**

Our findings suggest that HES 130/0.4 administered as CRI does not cause hypocoagulability in hypoalbuminemic dogs. A trend toward hypercoagulability, probably related to the underlying diseases, was observed in group 2 at T1.

Although all dogs were hyoalbuminemic, only 7/20 were hypercoagulable at T0, confirming the lack of correlation between albumin level and prothrombotic state.

## Background

Hydroxyethyl starch (HES) comprises a group of synthetic polymers routinely used in veterinary anesthesia and critical care medicine to maintain blood volume, counteract anesthesia-induced hypotension, to resuscitate patients with hypovolemic shock or sepsis, and to support intravascular colloid osmotic pressure (COP) during hypoalbuminemic states [1, 2]. HES can be administered as a bolus or as a constant rate infusion (CRI) typically at a rate of 20–50 ml/Kg/day and 1–2 ml/kg/h, respectively [1–3], the doses are generally extrapolated from human literature [1, 2]. The physicochemical and pharmacological properties of HES, as well as its classification, depend on their mean molecular weight (MW), molar substitution (MS), and C2/C6 ratio [4]. The rate of degradation and elimination of HES depends on the MS and C2/C6 ratio, whit higher MS and C2/C6 ratio leading to a slower elimination and greater intravascular retention time and associated side effects [5]. To improve safety and pharmacological properties, newer third-generation starch products with reduced MW and MS have been developed [4] that have shorter half-life, improved pharmacokinetic and pharmacodynamic properties, and fewer side effects [6]. Nonetheless, controversy in human medicine and veterinary medicine persists concerning the safety of HES. Indeed, recent reports of side effects in people (e.g., acute kidney injury, tissue storage, and impaired primary and secondary hemostasis), led to a temporary suspension of HES products. Authorization was reinstated about a year later, together with new guidelines for contraindications in certain conditions (e.g., sepsis) [2].

Hypocoagulability and increased risk of bleeding after administration of HES in human patients is believed to be due to hemodilution and direct effects of HES on the hemostatic system. Plasma accumulation of starch macromolecules can lead to platelet dysfunction, decreasing expression and activation of the surface receptor GPIIb/IIIa, decreased concentration of circulating von Willebrand factor (vWf) and factor VIII, impaired factor XIII fibrin cross-linking, and enhanced fibrinolysis resulting in a weaker and smaller clot [4, 7].

In veterinary medicine, both in vitro [8–14] and in vivo [15–20] studies have demonstrated impairment of whole blood coagulation after the administration of HES. After blood dilution with both crystalloids and colloids, in vitro studies have found a dose-dependent decrease in platelet function, an increase in prothrombin time (PT) and partial activated thromboplastin time (aPTT), and changes in viscoelastic parameters toward hypocoagulability (thromboelastometry or thromboelastography), with more severe impairment observed after dilution with HES [8–14]. Three in vivo studies have assessed only platelet function, whereas others studies have evaluated secondary hemostasis or whole blood coagulation following bolus administration of HES (doses ranging from 10 to 40 ml/kg in approximately 30 min), demonstrating hypocoagulability in healthy dogs or dogs affected by experimentally induced disease [15–20].

Viscoelastic techniques, such as thromboelastometric analysis (ROTEM), evaluate coagulation in whole blood samples. Since they take into account plasmatic and cellular elements, they better reflect the cell-based model of hemostasis, that describes coagulation as a dynamic process in which plasmatic coagulation factors interact with the cell surface [21, 22]. Rotational thromboelastometry use several reagents in different profiles to measure the kinetics of clot formation (time required for clot formation), the mechanical properties (clot strength), and the time required for clot dissolution (fibrinolysis) [22, 23]. It is the most useful tool to assess the presence of a hypercoagulable state, as compared with standard coagulation assays (PT, aPTT, fibrin degradation products and D-dimers) [22, 23].

To date, there are no published studies investigating the effects of HES 130/0.4 on whole blood hemostasis after its administration as a CRI in dogs with naturally occurring disease. Hypoalbuminemic dogs are a type of patients in which natural or synthetic colloids are frequently used to support the COP. These patients have also a high risk to form venous and arterial thrombi, and some studies have identified by means of ROTEM or thromboelastography (ROTEG) a hypercoagulable condition in dogs with protein losing enteropathy (PLE) or nephropathy (PLN) [24–27]. The hypothesized mechanism at baseline of this prothrombotic condition is a loss of proteins (from the kidney or the gut), which implies the loss of anticoagulant factors such as antithrombin [28–30]. In human medicine multiple mechanisms are involved in the development of hypercoagulability in case of hypoalbuminemia, including spontaneous platelet aggregation, increase in coagulation factors, hypofibrinolysis, decrease in antithrombin and protein C [31, 32].

The first aim of the present study was therefore to evaluate the effects of HES 130/0.4 administered as a CRI (at dose of 1 ml/Kg/h and 2 ml/Kg/h) on hemostasis in hypoalbuminemic dogs, by means of ROTEM. The hypothesis was that HES administration will cause a hypocoagulability state.

The second aim was to determine how many hypoalbuminemic dogs were hypercoagulable at presentation.

## Methods

This randomized, clinical prospective trial was performed on client-owned dogs. The protocol was approved by the Bioethics Committee of the author’s University. The dog owners were informed about the methods and aims of the study and gave their written informed consent.

### Animals and study design

The dogs were selected from patients admitted to our Veterinary Teaching Hospital for hospitalization. The inclusion criteria were: hypoalbuminemia (albumin < 2 g/dl) regardless of underlying disease, normal perfusion parameters, to require intravenous fluid therapy due to their underlying disease (e.g., increased losses, anorexia, dehydration). All dogs underwent complete physical examination and perfusion parameters were assessed by clinical evaluation and non-invasive measurement of arterial blood pressure using Doppler (Model 811-B, Parks Medical Electronics Inc., Oregon, USA). Exclusion criteria were pulmonary disease, cardiac and liver failure, renal azotemia, pre-existing hypocoagulability, and history of non-steroidal anti-inflammatory drugs, steroidal drugs, artificial colloid or blood products administration in the 4 weeks prior to the study. Additional exclusion criteria were abnormal perfusion parameters (heart rate > 130 bpm, poor pulse quality, capillary refill time > 2 s or < 1 s, systolic blood pressure < 90 mmHg and venous lactate > 2 mmol/L) and serologic test positive to *Ehrlichia canis, Dirofilaria immitis*, *Borrelia burgdorferi*, *Anaplasma phagocytophilum*, *Anaplasma platys* and/or *Leishmania infantum*.

Blood samples were collected by atraumatic venipuncture of the jugular vein with a 20-gauge needle using minimum stasis. Samples that were difficult to obtain (e.g., venipuncture required numerous attempts, needle repositioning or interruption of blood flow into the tube) were discarded and blood collection was repeated from the contralateral jugular vein. After blood collection, cell blood count (CBC) (ADVIA® 120 Hematology, Siemens Healthcare Diagnostics, Tarrytown, NY, USA), serum concentration of albumin (ILAB 300 plus, Clinical Chemistry System, Instrumentation Laboratories, Milan, Italy), total solid and packed cell volume were measured according to the study protocol. Other specific analyses were performed to assess the cause of hypoalbuminemia, as needed on a case-by-case basis.

Coagulation was assessed by standard coagulation profile [prothrombin time (PT), activated partial thromboplastin time (aPTT), and fibrinogen] (Coagulometer StART, Diagnostica Stago, Parsippany, NJ, USA), and by ROTEM analysis, (ROTEM®, TEM Innovations GmbH, Munich, Germany) for evaluating in-TEM®, ex-TEM®, and fib-TEM® profiles. Blood samples were divided into two tubes containing 3.2% buffered sodium citrate (Vacumed 3.2% buffered sodium citrate, FL Medical, Torreglia, Italy).

Thromboelastometric analyses were performed according to PROVETS guidelines [33, 34], and the analyses run for 60 min. For each blood sample, three thromboelastometric profiles were performed: in-TEM®, ex-TEM®, and fib-TEM® assay to evaluate the intrinsic and the extrinsic pathway, respectively, and assess the functional fibrinogen contribution to clot formation. For the in-TEM® profile, the blood sample was recalcified using the start-TEM® reagent and coagulation was activated with the specific reagent containing ellagic acid, whereas the ex-TEM® profile was activated by adding thromboplastin after recalcification. To obtain the fib-TEM® profile, coagulation was activated using thromboplastin in addition to a specific reagent containing a platelet inhibitor (cytochalasin D). The following variables were assessed for each profile: clotting time ([CT], s); clot formation time [(CFT) s]; maximum clot firmness [(MCF)] mm]; and α angle (α,°). Clotting time describes the time in seconds from clot initiation until the fibrin polymers are produced and the amplitude reaches 2 mm; this parameter is dependent on the concentration and activity of plasma coagulation factors [23]. Clot formation time, measured in seconds, is the time from initiation of clotting (2 mm) until an amplitude of 20 mm is reached [23]. Clot formation time and α angle provide information about the kinetics of clot formation and are predominantly affected by platelet count and function and fibrinogen concentration [23]. Maximum clot firmness represents the maximum amplitude reached by the clot, reflects maximal clot strength and stability, and is affected by fibrinogen, platelet count and function (except in the fib-TEM® profile where platelet are inhibited), thrombin, FXIII, and hematocrit (Hct) [23]. Additional calculated parameter is platelet contribution to maximum clot elasticity (MCE _platelet_), which evaluates the platelet component to clot strength and is obtained as follows: MCE_platelet_ = MCE_extem_-MCE_fibtem_ [MCE = (MCF*100)/(100-MCF)] [35].

A ROTEM tracing indicating hypocoagulability is characterized by an increase in CT and CFT and a decrease in MCF and α angle, whereas a ROTEM tracing indicating hypercoagulability is characterized by a decrease in CT and CFT and an increase in MCF and α angle. ROTEM tracings were considered abnormal when more than one ROTEM value was above (α angle, MCF) or below (CT, CFT) our reference ranges (Table 1).

**Table 1:** ROTEM and standard coagulation profiles results, before and 24 h after hydroxyethyl starch 130/0.4 infusion

	GROUP 1 (1 ml / kg / h) *N* = 11		GROUP 2 (2 ml / kg / h) *N* = 9		Institutional reference ranges
ROTEM	T0	T1	T0	T1	
In-TEM®					
CT (s)	180 (132–268)	166 (61–209)	188 (112–279)	141 (108–240)	126–363 s
CFT (s)	63 (45–132)	59 (41–113)	73 (41–86)	58 * (38–81)	47–224 s
MCF (mm)	72 (56–80)	72 (58–88)	68 (62–77)	72 (62–78)	50–75 mm
α angle (°)	77 (65–81)	79 (69–82)	76 (73–82)	78 * (74–82)	55–81 °
Ex-TEM®					
CT (s)	44 (27–61)	45 (20–77)	46 (30–89)	40 (33–80)	29–92 s
CFT (s)	67 (38–159)	78 (49–132)	81 (51–96)	62 (49–92)	54–275 s
MCF (mm)	77 (60–82)	70 (64–83)	70 (66–81)	72 * (67–86)	36–73 mm
α angle (°)	76 (61–83)	76 (65–82)	74 (71–80)	77 * (73–81)	47–79 °
Fib-TEM®					
CT (s)	47 (31–107)	39 (21–57)	45 (29–92)	39 (32–80)	14–102 s
MCF (mm)	25 (7–38)	16 (8–46)	15 (9–30)	20 (14–78)	6–26 mm
α angle (°)	77,5 (70–86)	76 (30–86)	76,5 (67–81)	77 (71–84)	40–78 °
MCE_platelet_	294 (142–404)	211 (169–441)	206 (175–383)	235 (183–571)	50–235
Standard coagulation					
aPTT (s)	12.5 (9.6–15.4)	12.3 (8.7–16)	13.9 (12.1–16)	14.5 (11.7–16)	12–16 s
PT (s)	7.5 (6.6–9.6)	7.15 (6.6–9.9)	7.4 (6.2–10)	7.3 (6.3–10)	8–10 s
Fibrinogen (g/L)	3.5 (2.5–8.5)	3.6 (2.4–7.9)	3.4 (2.2–7.4)	4.1 (2.2–7.1)	1.5–4.50 (g/L)

Values are expressed as median (minimum-maximum)

Values of institutional reference ranges for ROTEM parameters are expressed as 95% confidence intervals^14^

*In-TEM®* intrinsic thromboelastometry pathway, *Ex-TEM®* extrinsic thromboelastometry pathway, *Fib-TEM®* functional fibrinogen, *CT* clotting time, *CFT* clot formation time, *MCF* maximum clot firmness, *PT* prothrombin time, *aPTT* activated partial thromboplastin time

* Indicates statistically significant differences between T0 and T1 (*P* < 0.05)

Serological tests for *Ehrlichia canis*, *Dirofilaria immitis*, *Borrelia burgdorferi*, *Anaplasma phagocytophilum*, *Anaplasma platys* (Snap 4DX, IDEXX Laboratories, Westbrook, ME) and *Leishmania infantum* (Snap Leishmania, IDEXX Laboratories, Westbrook, ME) were also carried out.

An intravenous catheter was inserted into the peripheral vein, and fluid therapy was started using lactated Ringer’s solution (Ringer’s lactate solution, Baxter S.p.A, Rome, Italy), to replace dehydration, maintenance, and ongoing losses, and HES 130/0.4 [Voluven, Fresenius Kabi Italia srl., Isola della Scala (VR), Italy] for COP support. Hydroxyethyl starch 130/0.4 was administered as a CRI for at least 24 h. Colloid treatment [1 ml/kg/h (group 1) or 2 ml/kg/h (group 2)] was randomly assigned via a computer-generated program (Microsoft Excel, Redmond, WA, USA). The dogs were assessed for body temperature, respiratory rate and perfusion parameters every 4 h. Blood samples were collected at baseline (T0), to assess all previously laboratory analysis described, and 24 h after start of infusion (T1) CBC, albumin, packed cell volume, total solid (TS), and coagulation tests were repeated.

### Statistical analysis

Data were collected and analyzed with the software Stata 14.2 (Stata Statistical Software, release 10, StataCorp LP, College Station, TX, USA). Normality of data was assessed using the Shapiro-Wilk test.

To evaluate changes in ROTEM parameters after administration of the two different doses of HES 130/0.4 (CRI of 1 and 2 ml/kg/h) at two time points (T0 vs. T1), a hierarchical linear mixed effects model was used, where the random effect is given by the individual subject. Bonferroni correction was applied to detect in which medium the difference was statistically significant. When the data did not meet the assumption of normality, the comparison was conducted with the sign test.

Analysis of the data showed that only some dogs were hypercoagulable and that many had been randomly assigned to group 1. Despite the small number of hypercoagulable animals, Friedman’s test was carried out and the population divided (independent of HES dose administered) into two subgroups: hypercoagulable dogs (group H) and not hypercoagulable dogs (group NH). This was done to detect differences in ROTEM parameters at the two time points (T0 vs. T1).

A value of *P* < 0.05 was considered significant.

## Results

Of the total of 25 adult dogs initially enrolled, 5 were excluded because of Leishmaniosis (*n* = 2), liver failure (*n* = 2), and hypocoagulability with an abnormal thromboelastometric tracing at T0 (*n* = 1). The study sample comprised 20 dogs: 11 received HES 130/0.4 as a CRI at 1 ml/kg/h (group 1) and 9 at 2 ml/kg/h (group 2) for at least 24 h. In group 1, the median age was 7 years (min 2-max 12) and the median body weight 27 kg (min 5-max 39); breeds included: Australian Shepherd (*n* = 1), Border Collie (*n* = 1), Rottweiler (*n* = 1), German Shepherd (*n* = 1), Beagle (*n* = 1), and mixed breed (*n* = 6). Seven were females (4 neutered and 3 intact) and 4 males (1 castrated and 3 intact). Dogs included were affected by acute protein losing enteropathy (*n* = 4), chronic protein losing enteropathy (*n* = 3), protein losing nephropathy (PLN) (*n* = 1), or chylothorax (*n* = 3).

In group 2, the median age was 7 years (min 2 – max 10) and the median body weight 17.8 kg (min 5 – max 44); breeds included: Rottweiler (*n* = 1), English Bulldog (*n* = 1), Pit bull (*n* = 1), longhaired Dachshund (*n* = 1), Labrador (*n* = 1), Jack Russell (*n* = 2), and mixed breed (*n* = 2). Six were females (4 neutered and 2 intact) and 3 intact males. Dogs included were affected by chronic protein losing enteropathy (*n* = 6), chylothorax (*n* = 2) or hypoadrenocorticism (*n* = 1).

There were no statistically significant differences in age, body weight, serum albumin concentration, ROTEM values, PT and fibrinogen concentration between the two groups at baseline (T0). The aPTT was slightly prolonged in group 2 at both T0 and T1 (β = 2.2, C.I. 95% and *P* = 0.027, respectively), but still within the reference range.

Comparison between PT, aPTT, and fibrinogen concentration showed no statistically significant within-group differences between the two time points (T0 versus T1) (Table 1). While no statistically significant within-group differences in ROTEM values (T0 vs. T1) were found in group 1; statistically significant differences were noted in group 2 at T1: a decrease in CFT (*P* = 0.04) and an increase in the α angle (*P* = 0.02) in the in-TEM® profile and an increase in MCF (*P* = 0.008) and the α angle (*P* = 0.01) in the ex-TEM® profile (Table 1), whereas no changes was identified in the fib-TEM® profile and in the MCE_platelet_. Nevertheless, there were no statistically significant differences in ROTEM parameters between the two groups at T1. In both groups, there was a statistically significant decrease in Hct (*P* = 0.007) at T1 (time-dependent reduction), but no statistically significant dose-dependent difference between the two dosages (Table 2). There was no statistically significant difference in platelet count at T1 in either group (Table 2). Finally, a statistically significant increase in TS values (*P* = 0.03) in group 2 was noted at T1, but no statistically significant differences in albumin concentration in either group (Table 2).

**Table 2:** Results of laboratory parameters, before and 24 h after hydroxyethyl starch 130/0.4 infusion

	GROUP 1 (1 ml/kg/h) *N* = 11		GROUP 2 (2 ml/kg/h) *N* = 9	
	T0	T1	T0	T1
Hematocrit (%)	40 (29–54)	39 * (25–53)	38 (25–50)	35 * (25–55)
Platelet count (x 10E09 cell/L)	366 (156–853)	340 (145–800)	397 (125–641)	422 (134–731)
Total Solid (g/L)	0.36 (0.3–0.55)	0.36 (0.25–0.54)	0.32 (0.25–0.5)	0.35 * (0.27–0.5)
Albumin (g/L)	0.16 (0.12–0.19)	0.16 (0.11–0.24)	0.15 (0.13–0.18)	0.14 (0.11–0.22)

Values are expressed as median (minimum-maximum)

*Indicates statistically significant differences between T0 and T1 (*P* < 0.05)

ROTEM tracings indicating a hypercoagulable state were observed in 7/20 dogs at T0 (5/11 in group 1 and 2/9 in group 2). (Table 3) There were no statistically significant changes from T0 to T1 in either subgroup H or subgroup NH. Analysis of single animals classified as hypercoagulable (subgroup H) showed that the fibrinogen level was outside normal limits in 6/7 dogs (median 6.02, min 3.20 and max 8.53), whereas all the dogs classified as not hypercoagulable (subgroup NH) had levels within the normal range (median 3.26, min 2.23 and max 4.43) (Table 3). In addition, the MCE_platelet_ was outside the normal range in all the hypercoagulable dogs (median 339, min 257 and max 404) and it was increased only in 3/13 classified as not hypercoagulable (median 205, min 142 and max 316) (Table 3).

**Table 3:** ROTEM results at baseline (*N* = 20)

T0	In-TEM®				Ex-TEM®				Fib-TEM®					
G 1 (*N* = 11)	CT	CFT	MCF	α angle	CT	CFT	MCF	α angle	CT	MCF	α angle	MCE platelet	Fib	Disease
**Dog 1**	216	85	72	73	43	56	*80*	*80*	107	*38*	73	*339*	3.2	APLE
Dog 2	132	104	75	73	43	159	64	61	56	119	25	144	3.5	APLE
Dog 3	142	54	70	79	39	67	69	76	45	11	70	210	3.2	CPLE
Dog 4	144	52	70	80	27	74	67	75	31	13	73	188	2.7	CPLE
Dog 5	180	132	56	65	61	143	60	62	51	7	ND	142	3.5	APLE
**Dog 6**	180	45	75	81	41	*38*	*79*	*83*	38	*34*	*86*	*325*	*6*.*2*	PLN
**Dog 7**	156	47	*80*	80	53	66	*81*	79	50	*28*	*83*	*387*	*6*	CPLE
Dog 8	163	63	75	77	44	83	77	75	41	16	78	*316*	4.4	CH
**Dog 9**	241	50	*79*	80	61	*47*	*82*	*81*	54	*34*	*79*	*404*	*8*.*5*	CH
Dog 10	268	103	62	69	48	107	68	74	47	14	77	196	3.3	CPLE
**Dog 11**	184	66	70	76	46	60	*77*	78	37	*29*	*80*	*294*	*5*.*3*	APLE
G 2 (*N* = 9)														
**Dog 12**	241	52	*77*	79	46	*51*	*82*	*80*	45	*30*	*80*	*383*	*7*.*4*	CPLE
Dog 13	279	86	68	73	48	82	69	73	46	14	67	206	2.2	CPLE
Dog 14	198	79	67	74	40	96	67	71	36	22	79	175	4.2	CPLE
Dog 15	196	51	71	79	43	60	73	78	46	15	76	*253*	2.7	CPLE
**Dog 16**	*119*	*41*	72	*82*	30	57	75	79	29	*30*	*81*	*257*	*4*.*6*	CH
Dog 17	188	73	67	75	59	89	69	73	60	15	75	205	3.5	CH
Dog 18	151	75	64	76	38	72	70	75	38	22	77	205	2.8	CPLE
Dog 19	112	57	73	78	56	81	73	74	31	9	ND	*260*	2.4	AD
Dog 20	133	80	62	74	89	86	66	73	92	14	69	178	3.4	CPLE

*G 1* Group 1 (1 ml/kg/h), *G 2* Group 2 (2 ml/kg /h), *In-TEM®* intrinsic thromboelastometry pathway, *Ex-TEM®* extrinsic thromboelastometry pathway, *Fib-TEM®* functional fibrinogen, *CT* clotting time (s), *CFT* clot formation time (s), *MCF* maximum clot firmness (mm), *MCE*_*platelet*_ platelets contribution to clot elasticity, *Fib* fibrinogen level (g/L), *AD* hypoadrenocorticism, *APLE* acute protein losing enteropathy, *CH* chylothorax, *PLN* protein losing nephropathy, *CPLE* protein losing enteropathy, *ND* not determined, italicize numbers indicate the hypercoagulable dogs at T0 (N=7) and are outside the institutional reference ranges. Institutional reference ranges: In-TEM® (CT, 126–363 s; CFT, 47–224 s; MCF, 50–75 mm; α angle, 55–81°), Ex-TEM® (CT, 29–92 s; CFT, 54–275 s; MCF, 36–73; α angle, 47–79°), Fib-TEM® (CT, 14–102 s; MCF, 6–26; α angle, 40–78°)^14^ and fibrinogen level (1.5–4.50 g/L)

## Discussion

The main finding of the present study was that a hypocoagulable state was not observed by either 1 or 2 ml/kg/h of HES 130/0.4 in dogs with hypoalbuminemia. In contrast to our hypothesis, ROTEM analysis at T1 revealed statistically significant differences in some parameters consistent with a trend toward a hypercoagulability state after CRI at 2 ml/kg/h. In particular, in the in-TEM® profile a decrease in CFT and an increase in α angle, and in the ex-TEM® profile an increase in both α angle and MCF was found (Table 1). No statistical significant differences were shown comparing the ROTEM values of group 1 and group 2 at T1, then we can exclude that these variations have been induced by the different dose used in the group 2, and affirm that the change could be time dependent. As the dogs in this study presented with various different diseases, it is possible that an inflammatory state in the group 2 progressed during the 24 h of observation from T0 to T1 and affected coagulation in a different way. Since there is a well-established link between inflammation and coagulation, it might be possible that the inflammatory processes impaired activation of pro- and anti-coagulant factors, fibrinolysis, and induced abnormalities in platelets and endothelial components, but in our study we have not looked into markers for inflammation and antithrombin level [36].

Other hematological factors are known to influence the ROTEM analysis and may have affected our results. The CFT, α angle, and MCF can be influenced by some sample features such as platelet count, fibrinogen concentration, and Hct [23]. Our results have not found any statistically significant difference in platelet count and fibrinogen concentration, whereas a statistically significant reduction in Hct, after 24 h of infusion, was noted in both groups at T1. Hematocrit can affect ROTEM results, leading to a hypocoagulable tracing when Hct is increase or a hypercoagulable tracing when it is decreased [37, 38]. Smith et al. [37] hypothesized that a whole blood sample with a decreased Hct has a greater concentration of coagulation factors (the plasma to erythrocyte ratio changes in the ROTEM cup), leading to an artifactual hypercoagulability tracing when the blood is analyzed with ROTEM, and vice versa in case of increased Hct [37, 38]. Although no statistically significant difference in variation of Hct between the two groups was found from T0 to T1, the trend toward hypercoagulability identified in group 2 could be partially explained by the lower Hct values reached in this group at T1 (T1: Group 1, 39% and Group 2, 35% of Hct).

Although the platelet count remained unchanged between T0 and T1 (group 2), authors hypothesized that an increase in platelet activity could have been present. Indeed, no alterations were identified in the fib-TEM® profile, where platelet activity was inhibited. ROTEM is not a specific tool to evaluate platelet function, but MCE_platelet_ could be used to assess the contribution of platelets activity to clot elasticity, thus eliminating the influence of fibrinogen. However, no difference was identified between T0 and T1 in MCE_platelet_, and then a platelet contribution to hypercoagulable trend could be excluded.

Our findings differ from those reported in previous in vitro and in vivo studies that evaluated the effects of HES 130/0.4 on hemostasis by means of viscoelastic techniques (ROTEM and ROTEG). In two in vitro studies, when whole blood samples were diluted with HES 130/0.4 (1:22, 1:9, 1:4), a hypocoagulable ROTEM tracing was found only at the highest dilution (1:4, mimicking in vivo administration of 30 ml/kg) [11, 14]. Whereas, an impairment of secondary hemostasis has been identified after 1:5.5 dilution (comparable to a fluid dose of 20 ml/Kg) using ROTEG analysis [12]. The results of in vitro studies cannot be directly extrapolated to predict in vivo results, as the effect that a HES solution may have on hemostasis is largely determined by its in vivo pharmacokinetics [39].

Only three in vivo studies evaluating changes in hemostasis following HES 130/0.4 administration, are currently available in literature. One evaluated platelet function, whereas the other two assessed viscoelastic properties of whole blood [17, 18, 20]. Gauthier et al. [17] found a significant prolongation of aPTT and a hypocoagulable ROTEG after administration of HES 130/0.4 (bolus of 40 ml/kg over 30 min) in healthy dogs and dogs with induced systemic inflammatory response syndrome [17]. Reutler et al. [20] detected by means of ROTEM an impairment of whole blood coagulation after administration of a single bolus of HES (15 ml/kg over 30–40 min) in dogs undergoing general anesthesia for arthroscopy or imaging studies [20]. Comparison between our study and those mentioned above is difficult, because of the differences in study populations, rate and volume infused. The dogs in the present study were affected by naturally occurring diseases that cause hypoalbuminemia, whereas the sample populations in the other studies were healthy, anesthetized dogs or dogs with experimentally induced disease. In addition, the chosen method of HES administration, implied different infusion rate; during bolus the total amount of dose (15 or 40 ml/kg) is infused over a short period of time, resulting in an increase in intravascular volume (especially in hemodynamically stable dogs) and a greater hemodilution as compared with the administration as a CRI, where the total volume (24 or 48 ml/Kg) is equally divided over 24 h. Moreover, different rates are associated with a diverse degree of tissue accumulation and elimination [5].

At the time of inclusion in this study, only 7/20 dogs had a ROTEM tracing indicating hypercoagulability, and defined as a ROTEM profile with more than one parameter outside the institutional reference range. The fibrinogen level was normal at T0 and the MCE_platelet_ altered in 3 patients in the NH group. Differently in the H group, the fibrinogen level and the MCE_platelet_ were outside the upper range in 6/7 and 7/7 animals, respectively (Table 3). These results indicate an influence of the fibrinogen amount on the ROTEM tracing and an increase in the platelet aggregation, which can partially explain the hypercoagulbility detected at T0. In literature has been reported that an increase in platelet activity is associated with hypoalbuminemia in dogs with PLN [30, 40, 41]. Since there were no statistical significant differences from T0 to T1 in group H and group NH, in ROTEM results and MCE_platelet_, we can excluded the possibility that the hypercoagulability trend, seen at T1 in the group 2, was affected by the hypercoagulable patients.

Using ROTEG analysis, recent studies in dogs affected by PLE and PLN have identified hypercoagulability in the majority of them [25–27]. Due to the differences in the studies populations and the criteria for assessing hypercoagulability, a comparison with our study is not possible [25–27]. Until today, no standardized definition of hypercoagulability by ROTEM analysis in dogs has been established, although the PROVETS guidelines were issued in the attempt to achieve conformity across studies using viscoelastic techniques [42]. As there is insufficient evidence to recommend a definition of hypercoagulability in companion animals, the definition is left to the authors’ discretion [42].

An increased risk of thromboembolic events has been reported for dogs affected by PLN or PLE. The underlying cause of hypercoagulability remains incompletely understood; potentially involved mechanisms are loss of antithrombin and increased platelet aggregation [30, 40, 41]. Since we did not measure antithrombin activity, we are unable to determine its influence on our ROTEM results. Although multiple mechanisms have been involved in hypercoagulable state [31, 32], the incidence of thromboembolism dramatically increases when serum albumin concentration is less than 2.0–2.5 g/dL, in human patients with PLN [31, 32]. In contrast, veterinary studies have shown that hypercoagulability identified with ROTEM analysis does not appear to be correlated with hypoalbuminemia [25–27], and only a weak correlation between serum albumin concentration and antithrombin activity has been identified in hypercoagulable dogs, indicating that albumin level cannot reliably predict it [25–27, 43, 44]. These findings highlight that the prothrombotic state in dogs is not related only to hypoalbuminemia but to other abnormalities as well.

Refractometric evaluation was used to measure the TS concentration. Our results showed a statistically significant increase in TS after 24 h (T1) of HES 130/0.4 as a CRI at 2 ml/kg/h (Group 2). Bumpus et al. [45] reported that after the addition of a large volume of hetastarch (after an in vitro dilution of 1:4 corresponding to in vivo 22 ml/kg) the refractometer reading of TS increased [45]. Although the in vitro dosage administered in the aforementioned study differs from dosages used in the study herein, the higher dose of HES used in the group 2 may have interfered with the refractometric reading. Furthermore, the refractometer reading of HES 130/0.4 is 4.5 mg/dl, the same as that of the products used in the study by Bumpus et al. [45].

This study has several limitations. The small sample size, limits the ability to generalize the results obtained. Moreover, the clinicians were not blinded to the HES doses administered, which could have introduced a bias. The study population included dogs with diverse diseases, which might have several degrees of inflammation and different effect on hemostasis. In addition, we did not have a control group without treatment or treated only with a CRI of isotonic crystalloids, to determine whether the changes in hemostasis could be consequent to the progression of disease/inflammation or also related to the crystalloid infusion. In this regard, because the total amount of 24-h administration of crystalloids solution was not recorded, the influence of this variable could not be assessed.

Further studies on larger samples of dogs with naturally occurring disease causing hypoalbuminemia are needed to create disease categories and take into account the amount of infused crystalloid.

The assessment of HES 130/0.4 pharmacokinetics in dog could allow understanding the amount of its accumulation and the rate of elimination, especially when the colloid is administered as a CRI. In this context it would be important to evaluate the risks and benefits associated with HES therapy and its efficacy in providing oncotic support.

Another important field of research is to investigate the prevalence of hypercoagulability in hypoalbuminemic dogs, to better understand the impact of the underlying disease on the prothrombotic state and to correctly identify which hypoalbuminemic dogs could really benefit from anticoagulant therapy. Finally, further research for investigate the contribution of platelet function to hypercoagulable state, by means specific tools such as the PFA-100 or platelet aggregometer, would be interesting.

## Conclusion

Our findings suggest that CRI of HES 130/0.4 (1–2 ml/kg/h over 24 h) does not cause hypocoagulability in hypoalbuminemic dogs. A trend toward hypercoagulability, probably related to the underlying disease and associated degree of inflammation, was noted in group 2 at T1. Although all dogs were hyoalbuminemic, only 7/20 were hypercoagulable at T0, confirming the lack of correlation between albumin level and prothrombotic state.

## Abbreviations

aPTT: Activated partial thromboplastin time
CFT: Clot formation time
COP: Colloid osmotic pressure
CRI: Constant rate infusion
CT: Clotting time
HES: Hydroxyethyl starch
MCE: Maximum clot elasticity
MCEplatelet: Platelets contribution to clot elasticity
MCF: Maximum clot firmness
PLE: Protein losing enteropathy
PLN: Protein losing nephropathy
PT: Prothrombin time
ROTEG: Thromboelastography
ROTEM: Thromboelastometery
